# Bone morphogenetic protein 7 derived from DPP4^+^ cells in beige fat ameliorates age-associated metabolic dysfunction

**DOI:** 10.1093/lifemedi/lnad025

**Published:** 2023-07-04

**Authors:** Mingwei Guo, Jun Zhang, Ying Ma, Xia Wu, Jing Yao, Dongmei Wang, Jin Qiu, Peng Lu, Banru Chen, Jiqiu Wang, Lingyan Xu, Xinran Ma

**Affiliations:** Shanghai Key Laboratory of Regulatory Biology, Institute of Biomedical Sciences and School of Life Sciences, East China Normal University, Shanghai 200241, China; Shanghai Key Laboratory of Regulatory Biology, Institute of Biomedical Sciences and School of Life Sciences, East China Normal University, Shanghai 200241, China; Shanghai Key Laboratory of Regulatory Biology, Institute of Biomedical Sciences and School of Life Sciences, East China Normal University, Shanghai 200241, China; Shanghai Key Laboratory of Regulatory Biology, Institute of Biomedical Sciences and School of Life Sciences, East China Normal University, Shanghai 200241, China; Shanghai Key Laboratory of Regulatory Biology, Institute of Biomedical Sciences and School of Life Sciences, East China Normal University, Shanghai 200241, China; Shanghai Key Laboratory of Regulatory Biology, Institute of Biomedical Sciences and School of Life Sciences, East China Normal University, Shanghai 200241, China; Shanghai Key Laboratory of Regulatory Biology, Institute of Biomedical Sciences and School of Life Sciences, East China Normal University, Shanghai 200241, China; Department of Endocrine and Metabolic Diseases, Shanghai Institute of Endocrine and Metabolic Diseases, Ruijin Hospital, Shanghai Jiao Tong University School of Medicine, Shanghai 200025, China; Department of Endocrinology, Shandong Provincial Hospital Affiliated to Shandong First Medical University, Jinan 250021, China; Department of Endocrine and Metabolic Diseases, Shanghai Institute of Endocrine and Metabolic Diseases, Ruijin Hospital, Shanghai Jiao Tong University School of Medicine, Shanghai 200025, China; Department of Endocrine and Metabolic Diseases, Shanghai Institute of Endocrine and Metabolic Diseases, Ruijin Hospital, Shanghai Jiao Tong University School of Medicine, Shanghai 200025, China; Shanghai Key Laboratory of Regulatory Biology, Institute of Biomedical Sciences and School of Life Sciences, East China Normal University, Shanghai 200241, China; Shanghai Key Laboratory of Regulatory Biology, Institute of Biomedical Sciences and School of Life Sciences, East China Normal University, Shanghai 200241, China; Shanghai Frontiers Science Center of Genome Editing and Cell Therapy, Shanghai Key Laboratory of Regulatory Biology and School of Life Sciences, East China Normal University, Shanghai 200241, China; Chongqing Key Laboratory of Precision Optics, Chongqing Institute of East China Normal University, Chongqing 401120, China

## Dear Editor,

Beige fat is a highly flexible adipose tissue. It is indistinguishable from white fat under basal state but is activated under thermal stresses such as cold stimuli, mild hyperthermia, and β-adrenergic signaling via a process called ‘browning of white fat’ to strongly promote energy expenditure and lipid/glucose metabolism. During aging, beige fat mass and functionality experience programmed loss, which contributes greatly toward the onset and development of age-associated metabolic dysfunction, including increased adiposity, insulin resistance, and hyperlipidemia, yet detailed mechanisms remain unclear [1].

The bone morphogenetic proteins (BMPs) regulate numerous physiological and pathological processes such as proliferation, differentiation, and morphogenesis [2]. Among them, bone morphogenetic protein 7 (BMP7) has been shown to induce adipogenesis and activation of brown and beige fat, thus protecting against diet-induced obesity and insulin resistance [3]. However, the cellular source of BMP7 secretion and its upstream regulators in beige fat, as well as its role in age-associated metabolic dysfunction remains unclear.

**Figure 1.**
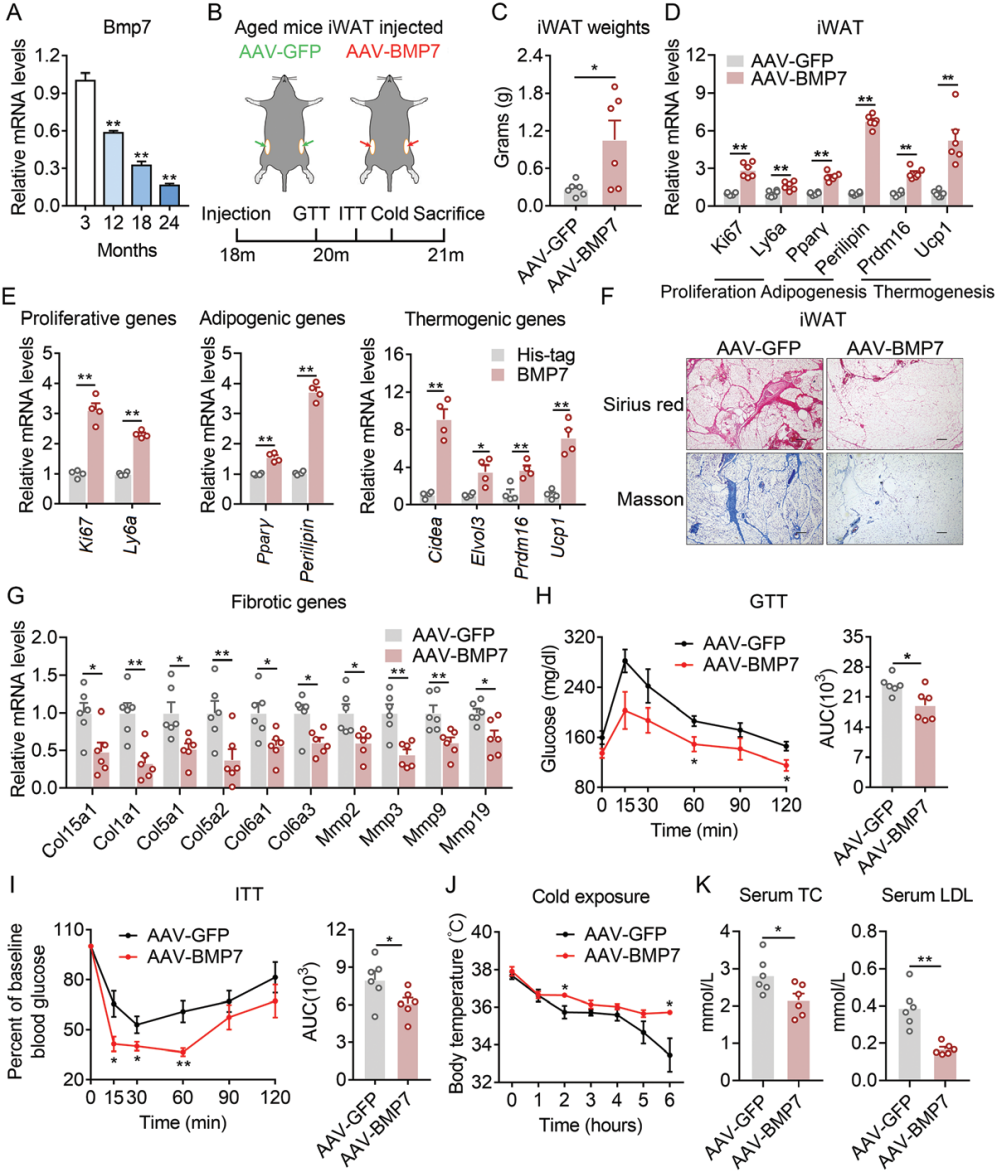
**AAV-mediated BMP7 delivery in iWAT ameliorates age-associated metabolic dysfunction.**(A) The relative mRNA level of BMP7 in iWAT of 3-, 12-, 18-, and 24-month-old mice. (B) Phenotypical and molecular analysis of aged mice (18 months old) locally administrated with AAV-GFP or AAV-BMP7 in iWAT for 8 weeks. *n* = 6 per group. (C–K) Diagram showing *in vivo* intervention strategy. (C) iWAT weights; (D) mRNA levels of proliferative, adipogenic, and thermogenic genes in iWAT administrated with AAV-GFP or AAV-BMP7; (E) mRNA levels of proliferative, adipogenic, and thermogenic genes of SVF treated with BMP7 recombinant protein or His-tag; (F) Histological analysis of sirius red and masson staining; (G) mRNA levels of fibrotic genes; (H) glucose tolerance test and area under the curve (AUC); (I) ITT and AUC; (J) Core body temperature hourly under cold exposure; (K) Serum parameters analysis for TC and low-density lipoprotein cholesterol (LDL-C). Data are presented as mean ± SEM and *P < 0.05, **P < 0.01 compared with a control group. Scale bar, 100 μm.

In the present study, we found that BMP7 mRNA levels feature an age-dependent decrease, suggesting it may involve in the alteration of beige fat function in the aging scenario (Fig. 1A). We then replenished BMP7 in beige adipocytes of 18-month-old aged mice by local injection of AAV-BMP7 into bilateral inguinal fat (iWAT), a fat type enriched with beige adipocytes (Fig. 1B). Local BMP7 overexpression was successfully achieved and maintained, accompanied with downstream p-p38 and p-SMAD1/5/8 signaling activation (Fig. S1A and S1B). BMP7 overexpression resulted in a sharp increase in iWAT weights in AAV-BMP7 mice compared to AAV-GFP mice, without overt change in body weights (Figs. 1C and S1C). Besides, BMP7 mRNA levels were positively correlated with iWAT weights in these mice (Fig. S1D). Detailed molecular analysis revealed that BMP7 overexpression in iWAT significantly induced proliferative, adipogenic, and thermogenic genes expression (Fig. 1D). Consistent with that, when preadipocytes isolated from a stromal vascular fraction (SVF) of iWAT were treated with BMP7 recombinant protein, preadipocyte proliferation was dramatically increased as evident by enhancement in *Ki67* and *Ly6a* expression, total cell number, and proliferating cell number (Figs. 1E and S1E-F). Moreover, BMP7 administration along the differentiation process of primary adipocytes enhanced adipogenesis as shown by elevated adipogenic genes expressions such as *Pparγ* and *Perilipin* and stronger oil red staining (Figs. 1E and S1G), while treatment of mature adipocytes with BMP7 dramatically increased thermogenic genes such as *Ucp1*, *Cidea*, *Elovl3*, and *Prdm16* (Fig. 1E). In addition, *in vivo* BMP7 administration in iWAT promoted preadipocyte proliferation and enhanced novel adipocytes formation, as shown by increased EdU staining and more small adipocytes in BMP7 group compared to controls (Fig. S1H).

In addition, consistent with a role of BMP7 in stimulating beige adipocyte formation and suppressing TGF-β signaling, which is critical for tissue fibrosis [4], we found that beige fat in aged mice treated with AAV-BMP7 showed largely improved age-associated fibrotic phenotype, accompanied with reduced expressions of collagens and MMPs (Fig. 1F and 1G). In the physiological level, we found that AAV-BMP7 treated mice exhibited improved insulin sensitivity as shown by glucose and insulin tolerance test (ITT), increased thermogenic capacity as revealed by cold exposure analysis, as well as reduced total cholesterol (TC) and low-density lipoprotein levels under serum parameter analysis, compared with AAV-GFP group mice (Fig. 1H–K). Taken together, both *in vivo* and *in vitro* data demonstrated that ectopic expression of BMP7 in beige fat of aged mice ameliorated age-associated metabolic dysfunction by inducing proliferative and adipogenic genes of preadipocytes and thermogenic genes of mature beige adipocytes.

**Figure 2.**
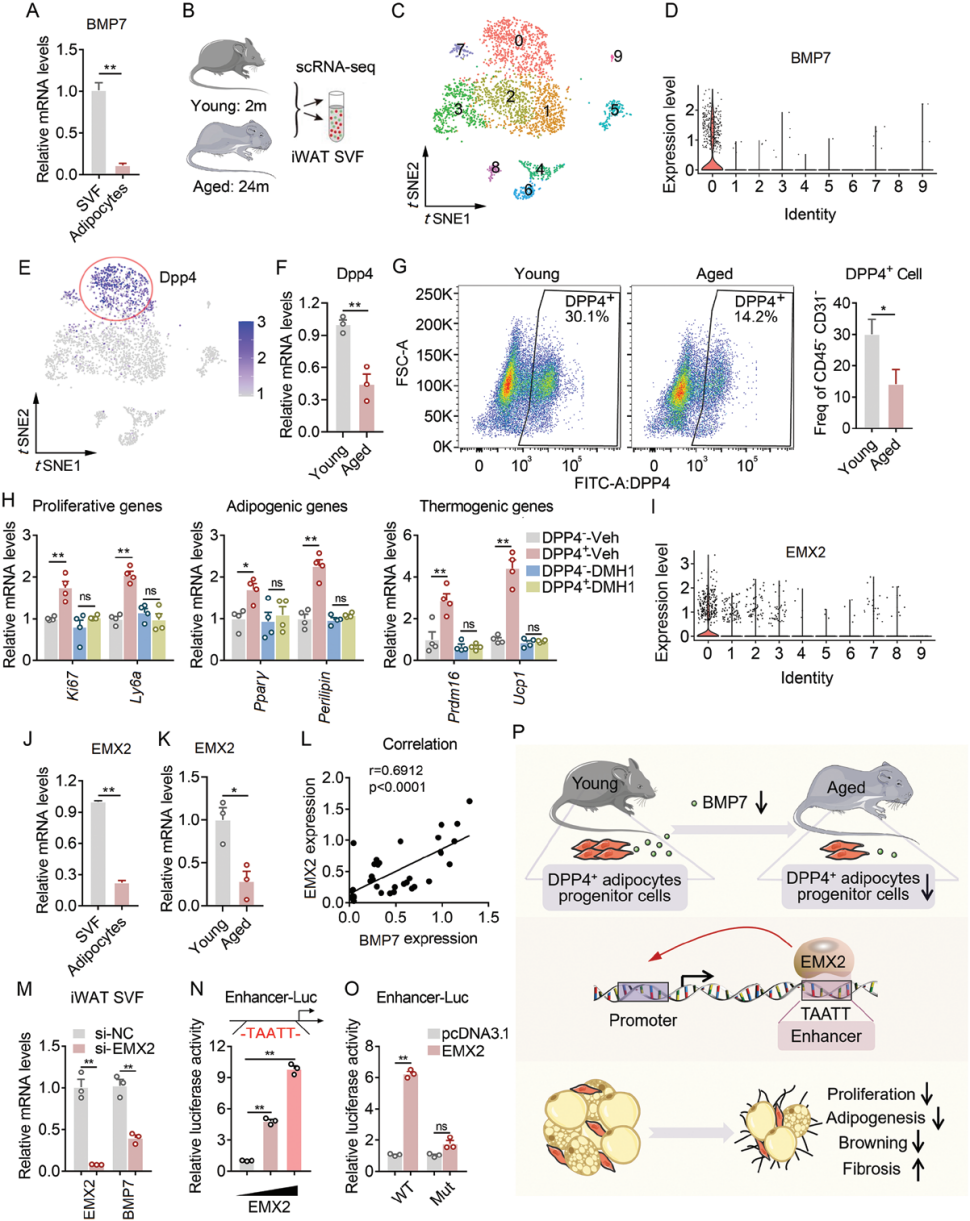
**Existence of EMX2–BMP7 axis in DPP4**^**+**^ **cells of beige fat.**(A) The relative mRNA level of BMP7 in SVF or adipocytes of iWAT from young mice. (B) Diagram showing single cell RNA sequencing in SVF of 2- and 24-month-old mice. (C) *t*-SNE plot of cell cluster 0–9. (D) Violin plot showing BMP7 specificity in cell clusters. (E) *t*-SNE plot showing DPP4 specificity in cell clusters. (F) The relative mRNA level of DPP4 in iWAT from 2- or 24-month-old mice. (G) Flow cytometry analysis of CD45^‐^/CD31^‐^DPP4^+^ cells from iWAT of 2- and 24-month-old mice. (H) mRNA levels of proliferative, adipogenic, and thermogenic genes of iWAT SVF co-cultured with medium from DPP4^+^ and DPP4^‐^ cells pretreated with vehicle or DMH1 for 24 h. (I) Violin plot showing EMX2 specificity in cell clusters. (J) The relative mRNA level of EMX2 in SVF or adipocytes of iWAT from 2-month-old mice. (K) The relative mRNA level of EMX2 in iWAT from 2- or 24-month-old mice. (L) The correlation of relative mRNA level between EMX2 and BMP7. (M) The relative mRNA levels of EMX2 and BMP7 in iWAT SVF were treated with siNC or siEMX2 for 24 h. (N) Luciferase assays show relative luciferase activities of EMX2 on BMP7 enhancer in a dose-dependent manner. (O) Luciferase assays showing relative luciferase activities of EMX2 on wildtype (WT) and mutated TAATT element on BMP7 enhancer. (P) Schematic diagram showing the existence of EMX2–BMP7 axis in DPP4^+^ cells of beige fat. Data are presented as mean ± SEM and *P < 0.05, **P < 0.01 compared with a control group. *n* = 6 per group for *in vivo* data and *n* = 3 for *in vitro* data.

Having shown that BMP7 is vital for the maintenance of beige fat functionality while its level declines during aging, we aim to understand the source of BMP7 secretion and its upstream regulator. Firstly, we found higher BMP7 expression in SVF than mature adipocytes (Fig. 2A). Considering the heterogenous nature of SVF, to further explore which cell population in SVF mainly responsible for BMP7 production, we comprehensively studied cell population and marker gene changes in iWAT SVF (depleting CD45^+^ cells) of 2- and 24-month-old mice via single cell RNA sequencing (scRNA-seq) (Fig. 2B). Single cell atlases of subcutaneous fat tissues in murine and human have deciphered the heterogeneity of stromal cells [5]. We mapped composition of cell populations in iWAT according to *t*-SNE analysis (Fig. 2C). Of note, violin plot analysis showed that BMP7 is uniquely expressed in DPP4^+^ population (Fig. 2D and 2E). Importantly, BMP7 levels featured a specific decline in DPP4^+^ subgroup in aged mice compared to young controls (Fig. S2A). Pseudo-time analysis revealed that DPP4^+^ marked adipocyte progenitor cells were original source of other cell populations (Fig. S2B), which were in line with previous report that DPP4^+^ population in adipose tissue gives rise to committed ICAM1^+^ and CD142^+^ preadipocyte to form mature adipocytes [5]. Critically, we found that DPP4 levels and DPP4^+^ cell numbers were decreased in aged mice compared to young ones (Figs. 2F-G and S2C-D), which may underlie the reduction in BMP7 levels in beige fat during aging. We then further analyzed whether BMP7 from DPP4^+^ cell population is main mediator for proliferation and adipogenesis of SVF and thermogenesis of mature beige adipocytes with a paracrine manner. Similar to BMP7 recombinant protein treatment, co-culture experiments revealed that DPP4^+^ cells promoted proliferative and adipogenic genes in preadipocytes, as well as thermogenic genes in mature beige adipocytes (Fig. 2H), which were blunted by pretreatment with DMH1, an inhibitor of BMP7 receptor [6] (Fig. 2H). Overall, these data suggested that DPP4^+^ population is the main source of BMP7 production in beige fat and that DPP4^+^-originated BMP7 contributed to improvement in age-associated metabolic dysfunction.

In investigating the upstream regulator of BMP7 production in DPP4^+^ cells, we noticed that transcription factor EMX2 is also specifically expressed in DPP4^+^ cells (Fig. 2I) and exhibited a similar expression pattern as BMP7, since EMX2 expressions were also higher in SVF than mature adipocytes (Fig. 2J) and reduced during aging (Figs. 2K and S2E). Indeed, Pearson analysis indicated a close correlation of mRNA levels between EMX2 and BMP7 (Fig. 2L). EMX2 is a transcription factor critical for development by binding to TAATT (DNA-binding motif of EMX2) elements in enhancer to induce target gene transcription [7]. Of note, gene expression analysis showed that knockdown of EMX2 led to reduction of BMP7 levels (Fig. 2M). Via *in silico* analysis, we identified a putative TAATT element in BMP7 enhancer and luciferase assay indicated that EMX2 induced the transcriptional activity of a BMP7 enhancer-luc construct in a dose-dependent manner (Fig. 2N), while deleting TAATT element in enhancer region fully blunted this activation (Fig. 2O). Taken together, these data suggested that EMX2 regulates BMP7 transcription in DPP4^+^ cell populations.

In summary, we demonstrated that BMP7 is derived from DPP4^+^ cell in SVF compartment of beige fat and mediated paracrine crosstalk between DPP4^+^ cells and preadipocytes or mature adipocytes to induce proliferation, adipogenesis and thermogenesis for enhanced beige fat functionality and reduced adipose tissue fibrosis. Besides, we revealed that the upstream transcription factor EMX2 regulates BMP7 levels in DPP4^+^ cells for BMP7 transcriptional activation (Fig. 2P). Since DPP4^+^ progenitor cell population, along with BMP7 and EMX2, were all reduced in iWAT of aged mice, the present study provides a basis for enhancing EMX2–BMP7 axis and DPP4^+^ progenitor cells to improve age-associated impaired fat biology and metabolic dysfunction. It has to be noted that circulating BMP7 may also contribute to the adipose tissue biology. Indeed, BMP7 decline is associated with liver and renal diseases, as well as muscle dysfunction and sarcopenia, which normally happen during aging. Besides, BMP7 overexpression has been shown to ameliorate these diseases, which may potentially improve adipose tissue thermogenic remodeling [8].

We also examined BMP4, another well-established BMP family member regulating the browning of white fat [9] and found that similar to BMP7, BMP4 mRNA levels in iWAT were also declined in an age-dependent manner (Fig. S3A). Besides, BMP4 was dominantly expressed in SVF compared with mature adipocytes and decreased in aged mice (Fig. S3B and S3C). On the other hand, via scRNA-seq analysis, we found that BMP4 expression is ubiquitously expressed in cluster 0, 1, 2, 3, and 7 cell types (Fig. S3D and S3E). Future work to investigate other BMP family members in the setting of aging would be interesting for fully understanding adipose tissue aging. In addition, it has been recently shown that IL33 secreted from PDGFRβ^+^DPP4^+^ cells contribute to the formation of thermogenic fat in response to cold exposure [10]. Future analysis of these secreted factors would provide vital information to fully understand the crosstalk network in beige adipose tissues during aging.

## Research limitations

Our study systematically illustrated beige fat heterogeneity of stromal cells during aging. However, future work on DPP4^+^ cell transplant applications in aged mice would be useful to demonstrate its role in secreting BMP7 and combating age-associated metabolic dysfunction to achieve a better understanding of cellular network. In addition, further examination and comparison of the alterations of DPP4^+^ cell population as well as EMX2–BMP7 axis in beige fat of young and aged human are warranted for clinical purposes.

## Supplementary Material

lnad025_suppl_Supplementary_Material
