# Supplementary material for: Bone morphogenetic protein 7 derived from DPP4+ cells in beige fat ameliorates age-associated metabolic dysfunction
Source: Life Med. 2023 Jul 4;2(4):lnad025. doi: 10.1093/lifemedi/lnad025 (PMC11749456; doi:10.1093/lifemedi/lnad025)
Supplement: lnad025_suppl_Supplementary_Material [file lnad025_suppl_Supplementary_Material.docx]

**Supplementary for**

**BMP7 derived from DPP4^+^ cells in beige fat ameliorates age-associated metabolic dysfunction**

**Materials and Methods**

**Cell culture and siRNA transfection**

The iWAT SVF cells were cultured in Dulbecco’s Modified Eagle Medium (DMEM, Gibco 8118116) supplemented with 10% fetal bovine serum (FBS, Gibco 10099-141), 1% penicillin and streptomycin. Cells were maintained in an incubator at 37℃ with 5% CO_2_. siRNAs targeting Emx2 and non-targeting siRNA (siNC) were purchased from GenePharma. For *in vitro* siRNA delivery, undifferentiated iWAT SVF cells were cultured in 24-well plate and transfected with polymer/siRNA nanoparticles. Briefly, 1.5 μg siRNA was incubated with 30 μg F13-16 in 50 μL deionized water and then diluted by 200 μL FBS-free DMEM media. The final media containing F13-16/siRNA complexes were added into cell wells. Cells were replaced with fresh DMEM medium after 6 hours and collected after 24 hours for further analysis.

**CCK-8 and EdU analysis**

The iWAT SVF cells were cultured in 96 wells and treated with BMP7 recombinant protein for 24 h and analyzed with proliferative capacity with CCK-8 assay (C0043, Beyotime) and EdU Cell Proliferation Kit with Alexa Fluor 488 (C0071L, Beyotime) following manufacturers’ instructions. For acute *in vivo* Edu analysis in iWAT, 18-months old mice were subcutaneously injected with BMP7 recombinant protein or His-tag (2 mg/kg) and intraperitoneally co-injected with EdU (50 mg/kg) every day for three days.

**Adipocyte differentiation and Oil Red O Staining**

The iWAT SVF cells were cultured at 95% confluency and were induced to differentiate for 2 days with an adipogenic cocktail (0.5 mM IBMX, 1 μM Dexamethasone, 5 μg/mL Insulin, 1 μM Rosiglitazone) in DMEM medium containing 1% penicillin and streptomycin and 10% FBS. Two days after induction, cells were changed every 48 hours with adipocyte maintenance medium containing 5 μg/mL Insulin and 1 μM Rosiglitazone for 6 days to fully differentiated. BMP7 recombinant protein or control His-tag was treated in medium along the process. Lipid droplets were visualized by Oil red-O staining. Briefly, the differentiated adipocytes were washed with PBS and then fixed with 4% paraformaldehyde for 20 min. After washing three times with PBS, the cells were stained with Oil Red O solution for 20 min and then imaged under the microscope.

**Clones, transfections and luciferase assays**

The Emx2 gene was amplified from iWAT cDNA by PCR and was cloned into Fugw vector. Bmp7 enhancer spanning from +3303 to +4344 predicted by UCSC Genome Browser was amplified from genome DNA by PCR and cloned into pGL4.23 [luc2 minP] vector. The mutated enhancer (Mut) were generated with deletion of the indicated enhancer (TAATT, ranging from +3855 to +3860) on Bmp7 enhancer luciferase reporter using gene splicing by overlap extension PCR (SOE-PCR). The primers used for plasmids construction were listed in Table S1. HEK293T cells were transfected with indicated plasmids with EZ-trans transfection reagent (Life Ilab Biotechnology, C4058L1090). Luciferase activity was examined with dual luciferase system (Promega, TM040) and pRL-SV40 vector was used as an internal control reporter after plasmids transfection for 24 hours.

**Real-Time PCR**

Total RNA was extracted from tissues or cells using RNAiso Plus (Takara, 9108) following manufacturer’s instructions. 1 μg of total RNA was reverse transcribed into cDNA using the PrimeScript^TM^ RT Master Mix (TaKaRa, RR036A). Quantitative real-time PCR was performed with the Roche LightCycler 480 system (Roche) using SYBR Green Master mix (Yeasen, 11143ES50). The mRNA levels were calculated by the 2^-ΔΔCT^ method with the level of 36b4 as the internal control. The primers used for real-time PCR were listed in Table S1.

**Western blots**

iWAT tissues were freshly isolated and frozen at liquid nitrogen. Protein extraction was performed with RIPA buffer containing 50-mM Tris (pH 7.4), 150-mM NaCl, 1% Triton X-100, 1% sodium deoxycholate, 0.1% SDS, sodium orthovanadate, sodium fluoride, EDTA, leupeptin, supplemented with 1-mM PMSF, 10-mM DTT and 10-μM protein kinase inhibitor. The protein concentrations were quantified using a BCA protein quantification kit (Beyotime) and the samples from the SDS-PAGE gels were transferred to a membrane of nitrocellulose by electrophoretic transfer. Membranes were blocked with 5% skimmed milk for 1 h at room temperature and then incubated with anti-BMP7 (Santa Cruz Biotechnology, sc-53917), phosphorylated-p38 MAPK (Beyotime, AM063), phosphorylated SMAD 1/5/8 (Beyotime, AF1375) and anti-β-actin (Santa Cruz, sc-47778) antibodies. The membranes were washed with TBST three times and incubated with IRDye secondary antibodies (926-68071 and 926-322210) from LI-COR Biosciences (NE, USA). The protein bands were detected by Odyssey® CLX imaging system (LI-COR Biosciences, NE, USA).

**Animal assays**

C57BL/6J male mice were purchased from Shanghai Laboratory Animal Center (Shanghai, China). Mice were maintained in standard 12-hour light/12-hour dark cycle and had free access to food and water. All mouse studies were carried out following the guidelines approved by the Animal Ethics Committee of East China Normal University.

**AAV Virus Packaging and Local Injection into iWAT**

AAV-BMP7 and AAV-GFP virus were constructed and packaged by Hanbio Techonology Company (Shanghai, China). For local iWAT AAV delivery, 18-month-old male mice were randomized into AAV-BMP7 group (*n* = 6) or AAV-GFP group (*n* = 6) and bilateral iWATs were injected with AAV at the dose of 1×10^11^ vector genome (vg) in a final volume of 100 μL per iWAT. The mice were sacrificed and analyzed after 2-month intervention.

**Glucose and insulin tolerance tests (GTT and ITT)**

For insulin tolerance tests, mice received an intraperitoneal injection of insulin (0.75 U/kg, Sigma, I9287). For glucose tolerance tests, mice were fasted 6 hours and injected intraperitoneally with a glucose solution in PBS (1.5 g/kg, Sigma, G8270). Plasma glucose levels were measured from tail vein blood at 0, 15, 30, 60, 90 and 120 min after insulin or glucose injections, using an automatic glucometer (OneTouch Ultra, Johnson’s). Area under the curve (AUC) was calculated by GraphPad software.

**Cold tolerance test**

For cold exposure analysis, mice were individually caged and exposed to 4℃ for 6 h with free access to water. Core body temperature was measured and recorded at the beginning and hourly under cold exposure with a rectal thermometer (Braintree Scientific).

**Histological analysis**

The fresh fat tissues were fixed into 10% neutral formalin liquid and 5 μm paraffin sections were prepared for Hematoxylin and eosin staining (H&E), Sirius red and Masson staining. Tissue sections were photographed using Nikon camera.

**Flow cytometry and co-culture analysis**

Stromal vascular fraction (SVF) cells obtained from iWAT of 2- and 24-month-old mice. APC-Cy7 CD45 (BioLegend, #103115), PE CD31 (BioLegend, #102407) and FITC DPP4 (BioLegend, #137805) antibodies were used in flow cytometry (FACS), CD45^-^CD31^-^DPP4^+^ cells were analyzed using a BD FACS Canto II analyzer and collected on a BD FACS-Aria cell sorter. the FACS data were analyzed using FlowJo software. For the co-culture assays, the undifferentiated and differentiated iWAT cells were cultured with medium from DPP4^+^ and DPP4^-^ cells pretreated with vehicle or DMH1 (Tocris, Cat. No. 4126) for 24 hours for further analysis.

**Single cell RNA sequencing analysis**

SVFs from iWAT of 2- and 24-month-old mice were prepared. Mouse CD45 MicroBeads (Miltenyi, 130-052-301) were used for depletion of leukocytes from the prepared SVF cell suspension. CD45^-^ cells were reserved for the the single cell RNA sequencing (Sinotech Genomics). Seurat package (https://satijalab.org/seurat/, version 4.1.1) was used to analyze scRNA-seq data. After removing doublets and cells with low quality, the cells that expressed more than 200 genes (min.features = 200) and genes with transcripts detected in more than 3 cells (min.cells = 3) were used for further analysis. The top 2,000 highly variable genes were used for downstream analysis. Unsupervised clustering analysis was using a resolution of 0.5. The *t*-SNE plots and violin plots were generated by R (version 4.2.0). For the pseudotime analysis, Monocle R (version 2.0) package was performed on cell clusters (cluster 0-9). The scRNA-seq data was uploaded in GEO database (PRJNA930155).

**Statistical analysis**

All data in this study were presented as Mean ± SEM. The normalcy of data was examined by Shapiro-Wilk normality test. Two-tailed unpaired Student’s *t-*test were used for statistical comparisons between two groups. Two-way ANOVA with Bonferroni’s multiple comparisons was used for comparisons of multiple datasets. *P* < 0.05 was considered as statistically significant. The correlation analysis was performed with Pearson correlation analysis. All statistical analyses were performed using Prism 8 or SPSS software. The *P*-values were displayed as **p* < 0.05, ***p* < 0.01. ns, non-significant.

**
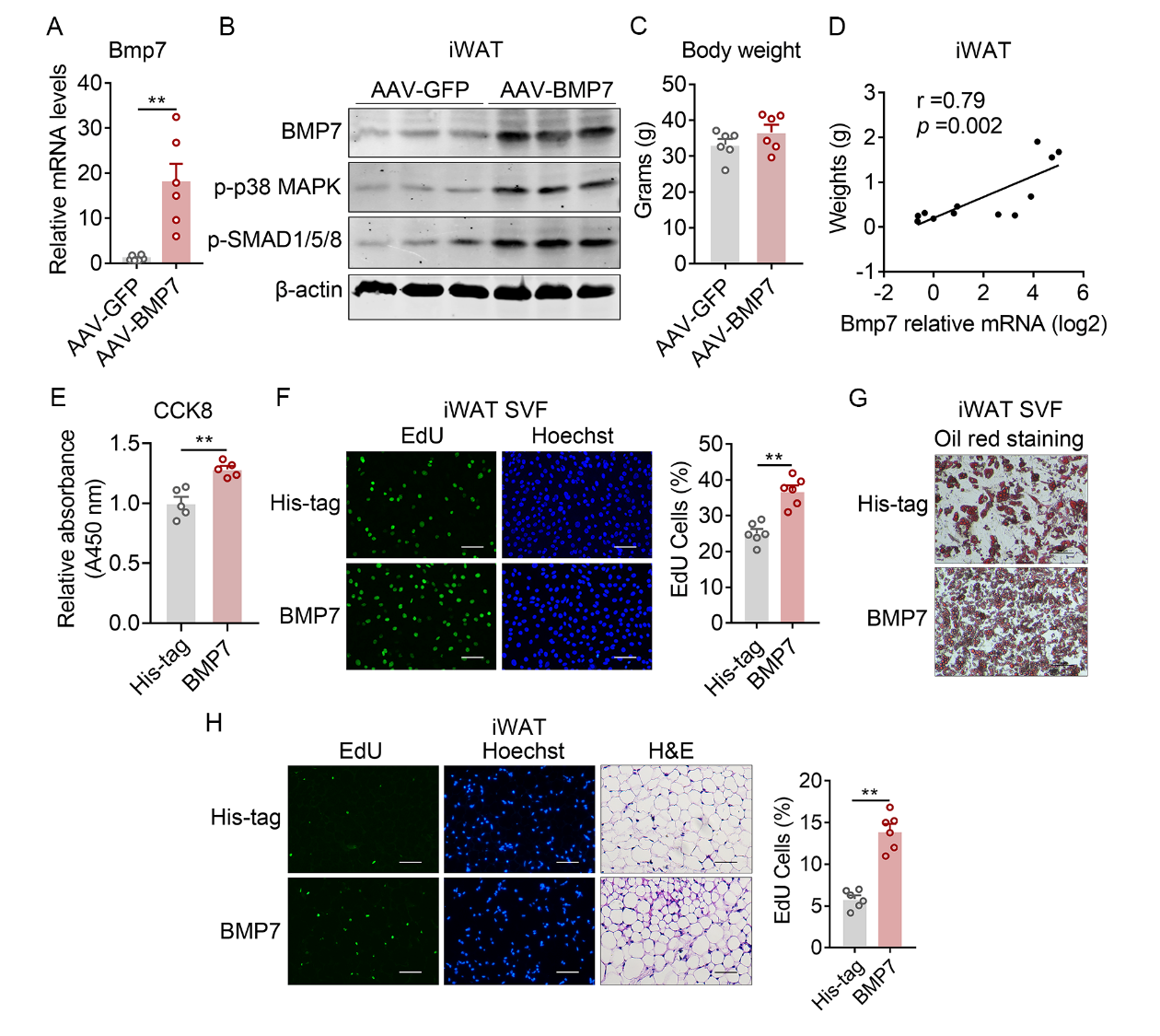
 Figure S1. BMP7 promotes proliferation and adipogenesis.**

(A-D) The molecular analysis and phenotype analysis in iWAT of aged mice (18 months old) locally administrated with AAV-GFP or AAV-BMP7 in iWAT for 8 weeks. *n* = 6 per group. (A) The relative mRNA levels of Bmp7; (B) The protein levels of BMP7, phosphorylated p38 MAPK (p-p38 MAPK) and phosphorylated SMAD1/5/8 (p-SMAD1/5/8); (C) The body weights; (D) The correlation analysis between iWAT weights and Bmp7 relative mRNA levels. (E, F) CCK8 and EdU analysis of SVF treated with BMP7 recombinant protein or His-tag for 24 hours. (G) Oil red staining of fully differentiated SVF treated with BMP7 or His-tag along with the differentiated process. (H) EdU analysis in iWAT of 18 months old mice were subcutaneously injected with BMP7 recombinant or His-tag protein (2 mg/kg) and intraperitoneally co-injected with EdU (50 mg/kg) every day for three days. Data are presented as mean ± SEM and **P* < 0.05, ***P* < 0.01 compared with control group. Scale bar, 100 μm.

**
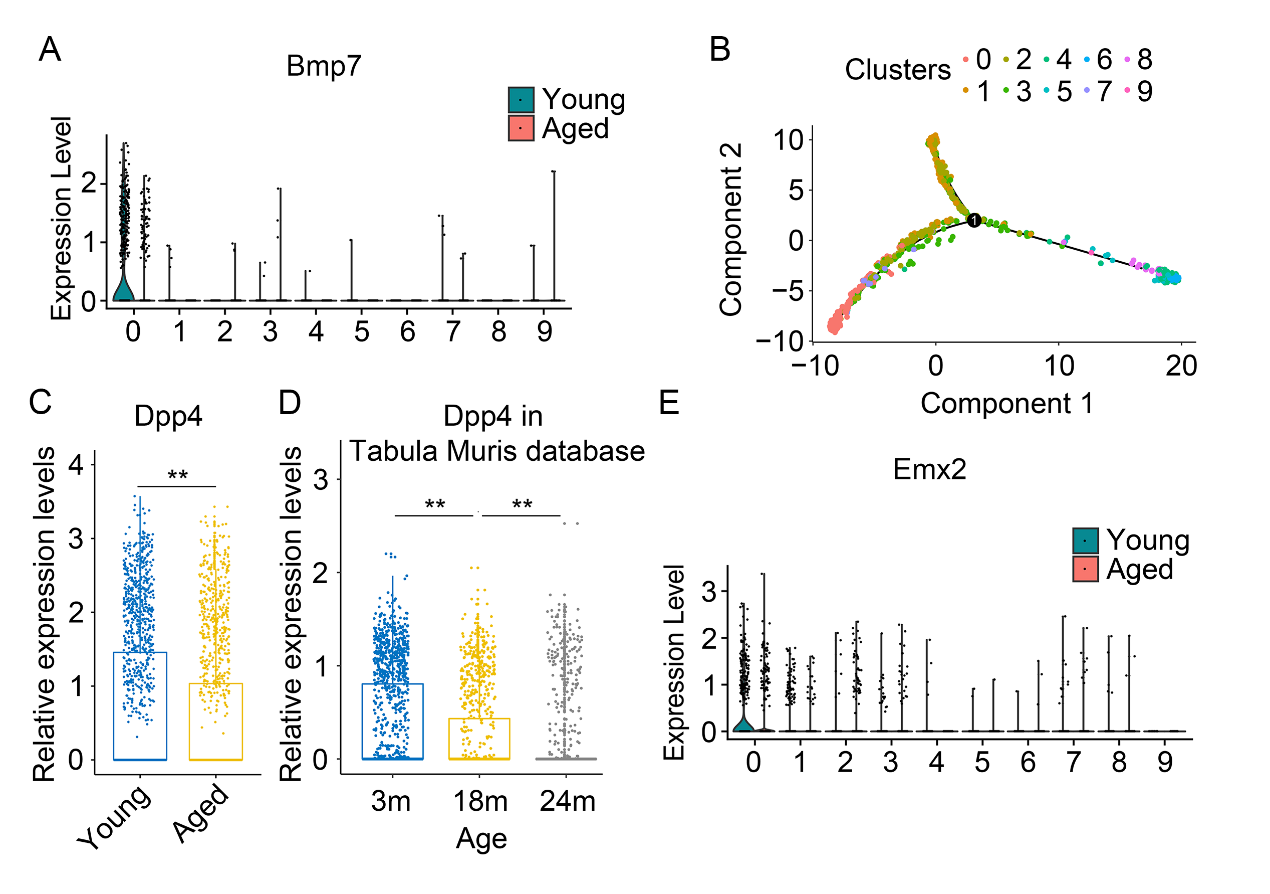
 Figure S2. Decline of Bmp7 and Emx2 levels in DPP4^+^ cells of beige fat.**

(A) Violin plot showing expression level of Bmp7 in DPP4^+^ cells of beige fat during aging. (B) Pseudo time analysis of cell cluster 0-9. (C, D) The relative expression level of Dpp4 from in-house scRNA-seq data and Tabula Muris database. (E) Violin plot showing expression level of Emx2 in DPP4^+^ cells of beige fat during aging. Data are presented as mean ± SEM and **P* < 0.05, ***P* < 0.01 compared with control group.

**
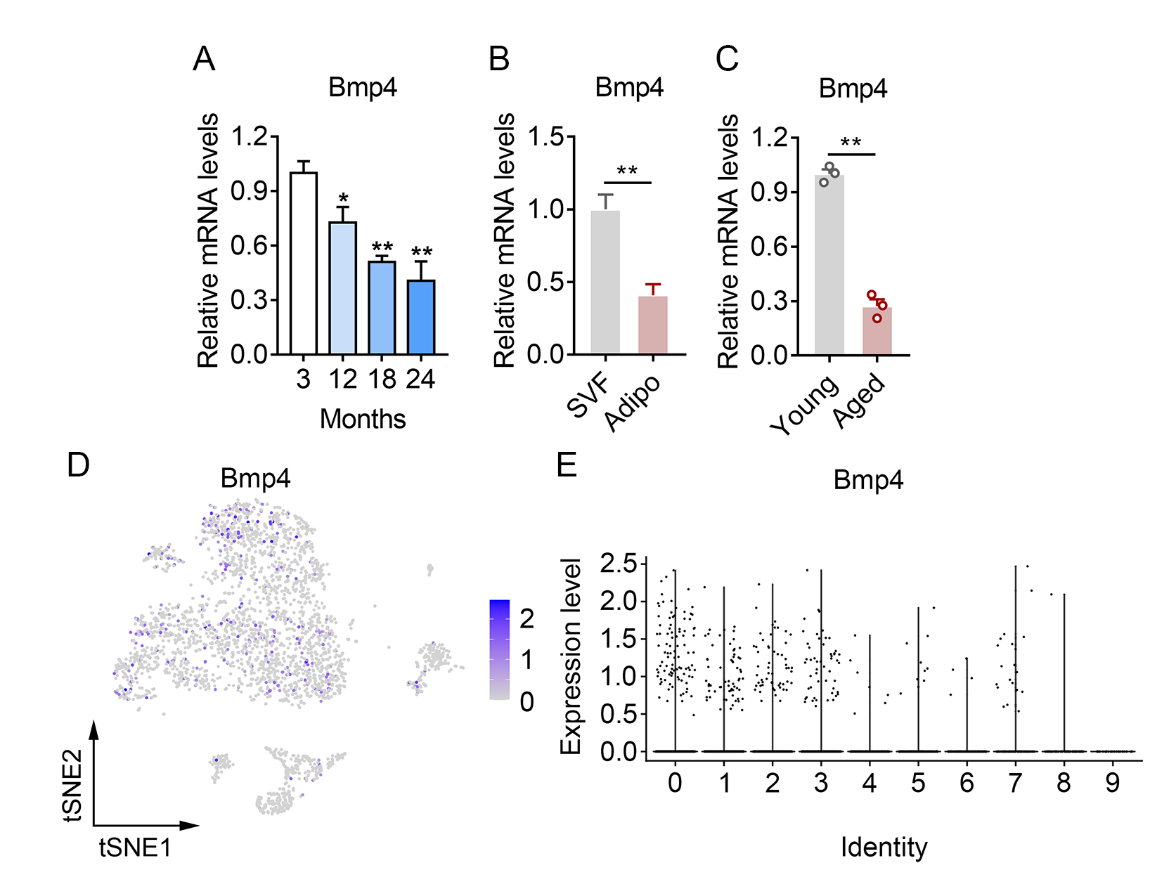
 Figure S3. Bmp4 expression pattern in beige fat of young and aged mice.**

(A) The relative mRNA level of Bmp4 in iWAT of 3-, 12-, 18- and 24-month-old mice. (B) The relative mRNA level of Bmp4 in SVF or adipocytes of iWAT from young mice. (C) The relative mRNA level of Bmp4 in iWAT SVF from 2- or 24-month-old mice. (D, E) *t*-SNE plot (D) and violin plot (E) showing Bmp4 specificity in cell clusters. Data are presented as mean ± SEM and **P* < 0.05, ***P* < 0.01 compared with control group.

**Table S1. PCR primers used in the study.**

| **Real-time PCR primers used in the study.** | | |
| --- | --- | --- |
| Gene name | Primer sequence (5′→3′) | |
| mKi67 | Forward | ACCGTGGAGTAGTTTATCTGGG |
|  | Reverse | TGTTTCCAGTCCGCTTACTTCT |
| mLy6a | Forward | GAGGCAGCAGTTATTGTGGAT |
|  | Reverse | CGTTGACCTTAGTACCCAGGA |
| mPparγ | Forward | GCATGGTGCCTTCGCTGA |
|  | Reverse | TGGCATCTCTGTGTCAACCATG |
| mPerilipin | Forward | GGGACCTGTGAGTGCTTCC |
|  | Reverse | GTATTGAAGAGCCGGGATCTTTT |
| mPrdm16 | Forward | CCACCAGCGAGGACTTCAC |
|  | Reverse | GGAGGACTCTCGTAGCTCGAA |
| mUcp1 | Forward | GGCCCTTGTAAACAACAAAATAC |
|  | Reverse | GGCAACAAGAGCTGACAGTAAAT |
| mCidea | Forward | TGACATTCATGGGATTGCAGAC |
|  | Reverse | CGAGCTGGATGTATGAGGGG |
| mElovl3 | Forward | TTCTCACGCGGGTTAAAAATGG |
|  | Reverse | TCTCGAAGTCATAGGGTTGCAT |
| mCol15a1 | Forward | CCCATTACCCTCGTCTGTGTC |
|  | Reverse | CTGAAGAAGGTCGGTGGGATG |
| mCol1a1 | Forward | CTGGCGGTTCAGGTCCAAT |
|  | Reverse | TTCCAGGCAATCCACGAGC |
| mCol5a1 | Forward | CTCCAACACCTCCAATCCAG |
|  | Reverse | GTCCTCCAATCCCCTCAAAG |
| mCol5a2 | Forward | ACAGGTGAAGTGGGATTCTCA |
|  | Reverse | CCATAGCACCCATTGGACCA |
| mCol6a1 | Forward | CTGCTGCTACAAGCCTGCT |
|  | Reverse | CCCCATAAGGTTTCAGCCTCA |
| mCol6a3 | Forward | GCTGCGGAATCACTTTGTGC |
|  | Reverse | CACCTTGACACCTTTCTGGGT |
| mMmp2 | Forward | GGACAAGTGGTCCGCGTAAA |
|  | Reverse | CCGACCGTTGAACAGGAAGG |
| mMmp3 | Forward | ACATGGAGACTTTGTCCCTTTTG |
|  | Reverse | TTGGCTGAGTGGTAGAGTCCC |
| mMmp9 | Forward | GCGTCGTGATCCCCACTTAC |
|  | Reverse | CAGGCCGAATAGGAGCGTC |
| mMmp19 | Forward | CCTGGTCCCATGCCAAACC |
|  | Reverse | CCCTTGAAAGCATAAGTCTTCCC |
| mDpp4 | Forward | ACCGTGGAAGGTTCTTCTGG |
|  | Reverse | CACAAAGAGTAGGACTTGACCC |
| mEmx2 | Forward | TCAGCTACGCCAATTCCAGTC |
|  | Reverse | ACCAAGTCCGGGTTGGAGTA |
| mBmp7 | Forward | ACGGACAGGGCTTCTCCTAC |
|  | Reverse | ATGGTGGTATCGAGGGTGGAA |
| m36b4 | Forward | AGATTCGGGATATGCTGTTGGC |
|  | Reverse | TCGGGTCCTAGACCAGTGTTC |
| **PCR primers for clone constructions in the study.** | | |
| Emx2 | Forward | TTTTTTGTTAGACAGGATCCGCCACCATGTTTCAGCCGGCGCCCAAGCG |
|  | Reverse | ATAAGCTTGATATCGAATTCTTAATCGTCTGAGGTCACATCTAT |
| WT-Bmp7-enhancer | Forward | TGGCCTAACTGGCCGGTACCGGTGCTCTAAGTACCACGCT |
|  | Reverse | ACCCTCTAGTGTCTAAGCTTCCAGTCCAGATCAAGAGAGCC |
| Mut-Bmp7-enhancer | Forward | TCAGACTCTTGAGTAGCTGGCAGGCCTCCATCCCAAGGCC |
|  | Reverse | GGCCTTGGGATGGAGGCCTGCCAGCTACTCAAGAGTCTGA |
